# Supplementary material for: Computing Solvation Free Energies of Small Molecules with Experimental Accuracy
Source: J Am Chem Soc. 2026 Jan 27;148(5):4928–37. doi: 10.1021/jacs.5c10940 (PMC12903862; doi:10.1021/jacs.5c10940)
Supplement: Supplementary file 1 [file ja5c10940_si_001.pdf]

Supporting Information for: Computing solvation free energies of small molecules with experimental accuracy

J. Harry Moore[†,†] Daniel J. Cole,[¶] and Gábor Csányi[*,†]

†*Engineering Laboratory, University of Cambridge, Cambridge, CB2 1PZ, UK*

‡*Ångström AI, 2325 3rd Street, San Francisco, CA 94107, USA*

¶*School of Natural and Environmental Sciences, Newcastle University, Newcastle upon Tyne NE1 7RU, UK*

E-mail: jhm72@cam.ac.uk

S1 Training and test set energy and force errors

Table S1: Route mean squared errors (RMSE) for training, validation and test set configurations for MACE-OFF24-SC. See previous work for details of the datasets[1]. Energy errors are in meV/atom, and force errors in meV/Å.

Dataset type	E RMSE	F RMSE	Rel. F RMSE %
Training	1.2	28.7	3.96
Validation	1.2	28.9	3.70
DES370K Dimers	2.1	15.2	3.23
DES370K Monomers	2.5	16.4	2.54
Dipeptides	2.5	22.9	3.25
PubChem	2.8	33.7	4.36
QMugs	2.3	35.5	3.53
Solvated Amino Acids	1.3	33.4	2.73
Water	1.9	23.6	3.72

S2 Octanol solvation free energy results

Table S2: Solvation free energies in kcal/mol in octanol for a series of organic solutes. GAFF2-ABCG2 values are taken from the literature.[2]

Molecule	SMILES	Experiment	Experiment Error	MACE	MACE Error	GAFF2/ABCG2
methanol	CO	-3.87	0.6	-3.87	0.09	-3.223
phthalimide	O=C1NC(=O)c2ccccc12	-11.18	0.6	-10.25	0.21	-10.945
methane	C	0.51	0.6	0.26	0.15	0.64
thiophene	c1ccsc1	-3.89	0.6	-3.05	0.11	-3.807
benzonitrile	N#Cc1ccccc1	-6.09	0.6	-6.56	0.15	-6.169
caffeine	Cn1cnc2c1c(=O)n(C)c(=O)n2C	-12.54	0.6	-11.73	0.17	-15.708
bromomethane	CBr	-2.43	0.6	-1.58	0.2	-2.264
o-cresol	Cc1ccccc1O	-8.49	0.6	-8.95	0.16	-8.58
benzene	c1ccccc1	-3.72	0.6	-3.7	0.11	-3.121
acetone	CC(=O)C	-3.15	0.6	-3.26	0.19	-2.935

S3 FreeSolv hydration free energy results

Table S3: Solvation free energies in kcal/mol for MACE, OpenFF and GAFF on the FreeSolv subset compared to experiment.

Compound Name	Exp	MACE	GAFF	OpenFF	Exp Error	MACE Error	GAFF Error	OpenFF Error
cycloheptanol	-5.48	-6.44	-4.34	-4.59	0.60	0.18	0.03	0.19
ethylene	1.28	0.33	2.33	2.29	0.60	0.09	0.01	0.09
2-chloropyridine	-4.39	-3.77	-3.87	-3.81	0.60	0.14	0.03	0.16
2-methylpyrazine	-5.51	-4.65	-6.16	-6.55	0.60	0.15	0.03	0.18
pentan-2-one	-3.52	-4.64	-3.17	-3.15	0.60	0.15	0.03	0.20
3-ethylpyridine	-4.59	-5.78	-2.96	-3.28	0.60	0.24	0.03	0.17
diphenyl ether	-2.87	-3.93	-2.81	-3.23	0.69	0.27	0.03	0.19
naphthalen-1-ol	-7.67	-7.20	-7.14	-8.43	0.60	0.19	0.03	0.20
fluoromethane	-0.22	-0.90	0.88	0.93	0.60	0.13	0.01	0.10
phthalimide	-9.61	-8.49	-11.82	-11.73	0.50	0.15	0.03	0.22
propan-2-ol	-4.74	-5.44	-3.43	-4.00	0.60	0.14	0.02	0.16
pyridine-3-carbonitrile	-6.75	-7.82	-5.58	-5.93	0.60	0.17	0.03	0.17
1-(3-pyridyl)ethanone	-8.26	-7.52	-7.84	-7.09	0.60	0.20	0.03	0.20
cyclopentanol	-5.49	-5.76	-4.29	-4.32	0.60	0.15	0.03	0.17
4-ethylpyridine	-4.73	-5.62	-3.19	-4.29	0.60	0.15	0.03	0.17
1-(4-pyridyl)ethanone	-7.62	-7.30	-7.57	-7.42	0.60	0.32	0.03	0.21
methane	2.00	1.33	2.45	2.40	0.20	0.09	0.01	0.15
ethanol	-5.00	-5.83	-3.39	-4.98	0.60	0.16	0.02	0.12
phenol	-6.60	-6.29	-5.71	-6.36	0.20	0.13	0.03	0.15
ethane	1.83	1.02	2.46	1.92	0.60	0.12	0.01	0.14
methanol	-5.10	-5.61	-3.49	-5.09	0.60	0.16	0.02	0.17
N,N-dimethylbenzamide	-9.29	-8.49	-8.11	-7.93	0.60	0.27	0.03	0.21
N-methylacetamide	-10.00	-10.43	-8.28	-7.90	0.60	0.31	0.02	0.18
ethylene glycol	-9.30	-10.63	-7.27	-8.89	0.60	0.15	0.03	0.20
alachlor	-8.21	-7.27	-6.85	-8.28	0.29	0.26	0.05	0.25
chlorobenzene	-1.12	-0.48	-0.47	-1.06	0.60	0.15	0.02	0.15
methylsulfinylmethane	-9.28	-10.88	-8.24	-7.03	0.57	0.20	0.02	0.17
2-ethylpyridine	-4.33	-5.16	-3.31	-3.37	0.60	0.16	0.03	0.19
fenuron	-9.13	-10.39	-11.81	-10.81	1.93	0.17	0.04	0.23
2-ethylpyrazine	-5.45	-5.14	-5.81	-5.94	0.60	0.19	0.03	0.21
pebulate	-3.64	-3.81	-4.57	-4.13	1.93	0.24	0.04	0.23
1-(2-hydroxyethylamino)-9,10-anthraquinone	-14.21	-14.34	-13.60	-14.45	1.10	0.34	0.05	0.28
cyclohexanol	-5.46	-6.35	-4.18	-3.83	0.60	0.14	0.03	0.21
1,4-dioxane	-5.06	-5.94	-4.27	-3.58	0.60	0.16	0.02	0.16
butan-2-ol	-4.62	-5.22	-3.15	-3.37	0.60	0.18	0.03	0.17
methylsulfanylethane	-1.5	-2.04	0.39	0.49	0.2	0.15	0.02	0.14

S4 Switching points

Dimer pair	Switching point / Å	Dimer pair	Switching point / Å
Br2	2.1499996	FO	1.2799997
BrCl	2.0199995	FP	1.5199996
BrF	1.6749996	FS	1.5249996
BrI	2.3149996	H2	0.6749997
BrN	1.6399996	HBr	1.3299996
BrO	1.6299996	HCl	1.2049997
BrP	2.0249996	HF	0.87999976
BrS	2.0049996	HI	1.4949995
C2	1.2149997	HN	0.9749997
CBr	1.7049996	HO	0.9199998
CCl	1.5549996	HP	1.3099995
CF	1.2149997	HS	1.2499996
CH	1.0349996	I2	2.480009
CI	1.8849996	IN	1.8049996
CN	1.1349998	IO	1.7749996
CO	1.0999999	IP	2.1949997
CP	1.4949996	IS	2.1649995
CS	1.4749997	N2	1.0699998
Cl2	1.8849995	NO	1.1149998
ClF	1.5499996	NP	1.4349997
ClI	2.1799996	NS	1.4399997
ClN	1.4899997	O2	1.1649997
ClO	1.4899997	OP	1.4299997
ClP	1.8899995	OS	1.4249997
ClS	1.8749995	P2	1.8049997
F2	1.3249997	PS	1.8149996
FI	1.8149996	S2	1.8049996
FN	1.2399998		

S5 Example of nonequilibrium switching

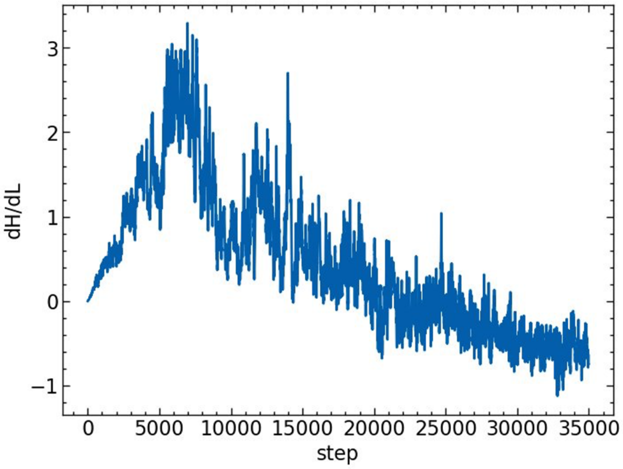

Figure S1: Example $\frac{dH}{d\lambda}$ plot for the solvation of methane in water over a 35 ps nonequilibrium switching trajectory.

S6 Free energy convergence plots

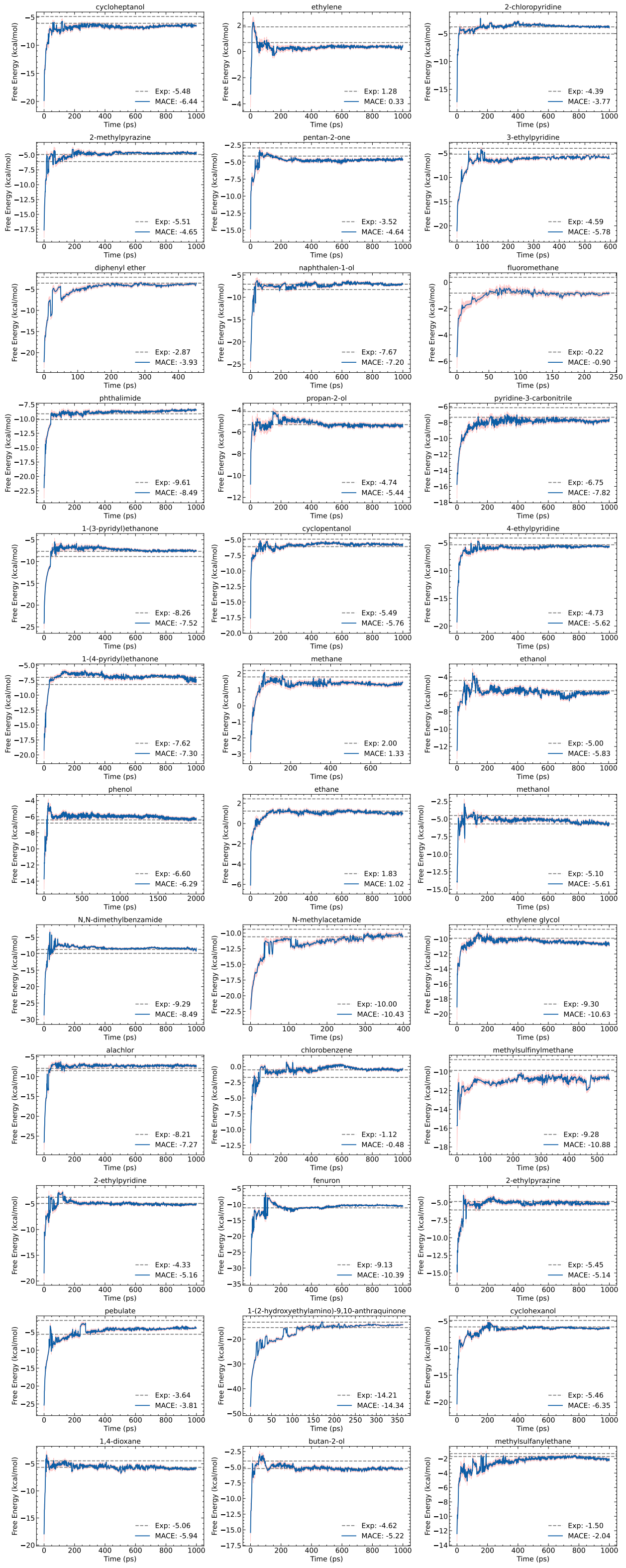

Figure S2: Convergence of hydration free energy calculations from FreeSolv benchmark.

S7 Sample replica exchange input file

```
#!/bin/bash
mpirun -np 8 mace-md -f /path/to/equilibrated/structure.pdb \
  --resname "LIG"\
  --output_dir /path/to/output/dir \
  --pressure 1.0 \
  --decouple \
  --restart \
  --dtype float32 \
  --log_level INFO \
  --run_type "repex" \
  --system_type "pure" \
  --interval 1 \
  --steps 1000 \
  --lambda_schedule="[0.   , 0.08, 0.1, 0.13,  0.15, 0.17, 0.20, 0.25,
    0.2825, 0.385 , 0.4875, 0.59  , 0.6925, 0.795 , 0.8975, 1.]"\
  --model_path "/path/to/mace/model" \
  --steps_per_iter 1000 \
```

Listing 1: Script to run 1 ns of replica exchange simulation via the MACE-MD code

S8 Example Absolv run script

```
import pathlib
import sys
import openmm.unit
import openff.toolkit
from absolv import config, runner
import absolv

smiles = sys.argv[1]
temperature=298.15 * openmm.unit.kelvin
pressure=1.0 * openmm.unit.atmosphere

system=absolv.config.System(
    solutes={smiles: 1}, solvent_a=None, solvent_b={"O": 1500}
)

alchemical_protocol_a=absolv.config.EquilibriumProtocol(
    lambda_sterics=[
        1.00, 1.00, 1.00, 1.00, 1.00, 0.95, 0.90, 0.80, 0.70, 0.60, 0.50, 0.40,
        0.35, 0.30, 0.25, 0.20, 0.15, 0.10, 0.05, 0.00,
    ],
    lambda_electrostatics=[
        1.00, 0.75, 0.50, 0.25, 0.00, 0.00, 0.00, 0.00, 0.00, 0.00, 0.00, 0.00,
        0.00, 0.00, 0.00, 0.00, 0.00, 0.00, 0.00, 0.00,
    ]
)
alchemical_protocol_b=absolv.config.EquilibriumProtocol(
    lambda_sterics=[
```

```
        1.00, 1.00, 1.00, 1.00, 1.00, 0.95, 0.90, 0.80, 0.70, 0.60, 0.50,
    0.40,
        0.35, 0.30, 0.25, 0.20, 0.15, 0.10, 0.05, 0.00,
    ],
    lambda_electrostatics=[
        1.00, 0.75, 0.50, 0.25, 0.00, 0.00, 0.00, 0.00, 0.00, 0.00, 0.00,
    0.00,
        0.00, 0.00, 0.00, 0.00, 0.00, 0.00, 0.00, 0.00,
    ]
)

config = absolv.config.Config(
    temperature=temperature,
    pressure=pressure,
    alchemical_protocol_a=alchemical_protocol_a,
    alchemical_protocol_b=alchemical_protocol_b,
)

force_field = openff.toolkit.ForceField("openff-2.1.0.offxml")
prepared_system_a, prepared_system_b = absolv.runner.setup(system, config,
    force_field)

result = absolv.runner.run_eq(
    config, prepared_system_a, prepared_system_b, "CUDA"
)

path = pathlib.Path("results")
(path / "output.txt").write_text(result.model_dump_json(indent=2))
```

Listing 2: Script to run equilibrium hydration free energy calculation with openff-2.1.0.

S9 CHEMBL compounds

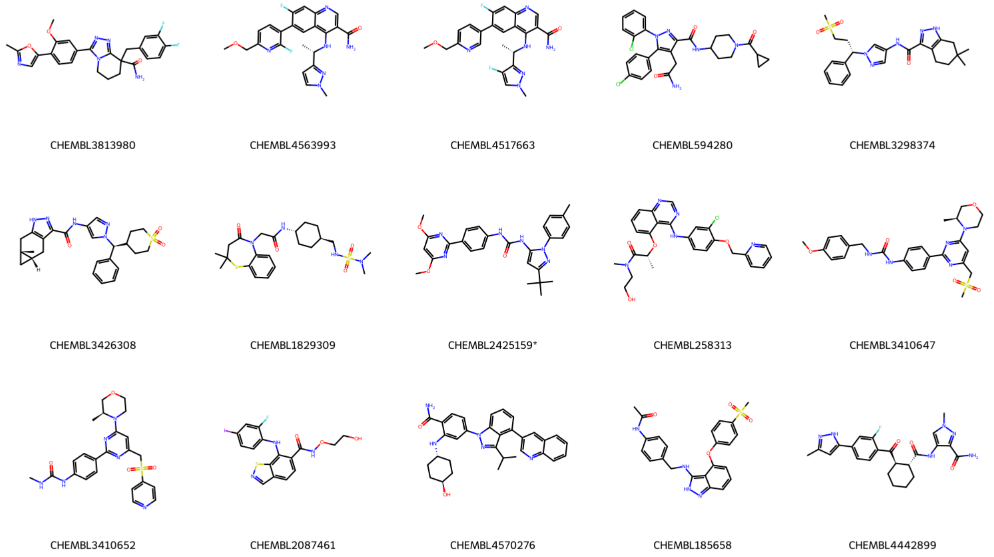

Figure S3: Structures of the CHEMBL compounds used for the logP calculations.

S10 Octanol Density

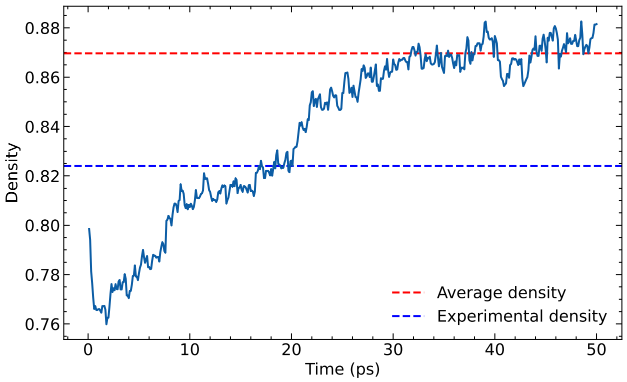

Figure S4: Density of liquid octanol simulated by `MACE-OFF24-SC`. Average density was calculated from the last 20 ps of the simulation

S11 Example GROMACS MDP file

```

title        = Absolute Solvation Free Energy in Octanol
integrator   = sd
nsteps       = 250000    ; 500 ps per window
dt           = 0.002
nstenergy    = 500
nstlog       = 500
nstxout-compressed = 500

continuation = yes
constraint_algorithm = lincs
constraints = h-bonds
lincs_iter  = 1
lincs_order = 4

nstlist          = 40
cutoff-scheme    = Verlet
ns_type          = grid
coulombtype      = PME
rcoulomb         = 1.2
rvdw             = 1.2
pbc              = xyz
disp_corr    = EnerPres

tc-grps      = System
tau_t        = 0.1
ref_t        = 298.15

pcoupl       = Parrinello-Rahman
pcoupltype   = isotropic
```

```
tau_p        = 2.0
ref_p        = 1.0
compressibility = 4.5e-5

; Free energy control
free_energy      = yes
init_lambda_state = 0   ; will be overwritten
delta_lambda     = 0
calc_lambda_neighbors = 1 ; calculate delta H to neighbors

; Lambda vectors
coul_lambdas = 0.00 0.14 0.29 0.43 0.57 0.71 0.86 1.00 1.00 1.00 1.00 1.00
    1.00 1.00 1.00 1.00 1.00 1.00 1.00 1.00 1.00 1.00 1.00 1.00
vdw_lambdas  = 0.00 0.00 0.00 0.00 0.00 0.00 0.00 0.00 0.06 0.12 0.19 0.25
    0.31 0.37 0.44 0.50 0.56 0.62 0.69 0.75 0.81 0.87 0.94 1.00

couple-moltype  = mol_009
couple-lambda0  = vdw-q
couple-lambda1  = none
couple-intramol = yes

sc-alpha     = 0.5
sc-power     = 1
sc-sigma     = 0.3
nstdhdl      = 100
```

Listing 3: mdp file for production GROMACS calculations for GAFF2/ABCG2 calculations

References

(1) Kovács, D. P.; Moore, J. H.; Browning, N. J.; Batatia, I.; Horton, J. T.; Pu, Y.; Kapil, V.; Witt, W. C.; Magdău, I.-B.; Cole, D. J.; Csányi, G. MACE-OFF: Short-Range Transferable Machine Learning Force Fields for Organic Molecules. *Journal of the American Chemical Society* **2025**, *147*, 17598–17611.

(2) He, X.; Man, V. H.; Yang, W.; Lee, T.-S.; Wang, J. ABCG2: A Milestone Charge Model for Accurate Solvation Free Energy Calculation. *Journal of Chemical Theory and Computation* **2025**, *21*, 3032–3043.
